# Membrane Lipids and Osmolytes Rearrangements Under Cell Wall Stress in *Aspergillus niger*

**DOI:** 10.3390/ijms262210888

**Published:** 2025-11-10

**Authors:** Elena A. Ianutsevich, Olga A. Danilova, Sofiya A. Saharova, Vera M. Tereshina

**Affiliations:** Winogradsky Institute of Microbiology, Research Center of Biotechnology of the Russian Academy of Sciences, 33, Bld. 2 Leninsky Ave., 119071 Moscow, Russiav.m.tereshina@inbox.ru (V.M.T.)

**Keywords:** cell wall stress, Congo red, calcofluor white, phosphatidic acids, polyols, chitin, glucan

## Abstract

The cell wall integrity pathway is activated in response to cell wall stress (CWS). The defense system in aspergilli employs three transcription factors—RlmA, MsnA, and CrzA—which also facilitate adaptation to various abiotic stressors and involve alterations in cytosolic osmolyte composition and membrane lipid profiles. However, their role in adaptation to CWS remains unclear. In *Aspergillus niger*, CWS induced by Congo red and calcofluor white caused a pronounced cessation of apical growth, accompanied by hyphal globular swelling and an increase in chitin and glucan content in the cell wall. Regarding the osmolyte composition, which predominantly consists of low levels of glycerol and mannitol, glycerol levels were reduced under CWS. Neither the composition nor the amounts of membrane and storage lipids changed following CWS; however, the degree of unsaturation of phospholipids increased due to a higher proportion of linolenic acid, potentially enhancing membrane fluidity. These minor rearrangements of membrane lipids and osmolytes do not confirm their involvement in the adaptation to CWS induced by Congo red and calcofluor white, contrary to previous assumptions based on studies of cell wall integrity pathways.

## 1. Introduction

The cell wall (CW) maintains the cell shape and integrity and is part of the cell envelope, serving as the first line of defense against various adverse environmental conditions, along with the plasma membrane [1]. The fungal CW is a dynamic and multifunctional structure responsible for biotic and abiotic interactions. It protects against osmotic and mechanical stresses and preserves the cell morphology [2]. It also serves as the site for numerous enzymes and acts as a matrix for surface proteins and glycoproteins involved in recognition, adhesion, and sexual interaction [3]. CW remodeling occurs throughout the life cycle and in response to stressors, nutrient deficiencies, damage, and the establishment of mutualistic or parasitic interactions [2].

Unlike yeast, the CW of filamentous fungi comprises a variety of glucans (β-1,3-, β-1,3/β-1,4-, β-1,6-, and α-1,3-glucans), chitin and chitosan, mannan and/or galactomannan, and glycoproteins [4]. Glucans and chitin are synthesized as linear polymers by their respective synthases associated with the plasma membrane. The predominant component of the fungal CW is β-1,3-glucan, which constitutes 30–80% of the CW by weight. It does not form microfibrils; it is a branched polymer with β-1,6 branches, adopts a triple helix structure, and serves as a matrix for protein attachment [5]. However, linear α-1,3-glucan is capable of forming microfibrils [6]. Chitin is a linear homopolymer of N-acetyl-D-glucosamine with β-1,4 bonds. The proportion of chitin in fungal CW varies from 2% (in yeast) to 30% (in filamentous fungi). Numerous hydrogen bonds form between the antiparallel chitin chains in the CW region, resulting in the formation of microfibrils or rodlets [5]. Chitin’s rigidity is due to its linear and antiparallel chains, making it one of the strongest biopolymers in nature. Chitin is localized as microfibrils in the inner layer of the CW and is covalently bound to flexible β-1,3-glucan chains. Glycoproteins are synthesized in the endoplasmic reticulum and Golgi apparatus and enter the CW region by secretion. Furthermore, through the action of crosslinking transglycosidases and glycosyl hydrolases, glucans, chitin, and glycoproteins covalently bind to form the three-dimensional structure of the CW. The arrangement of the rigid sections interspersed with the flexible regions appears to be a consistent feature of fungal CW, although the chemical composition can vary. For example, in *Aspergillus fumigatus*, chitin is tightly packed with α-1,3-glucan, forming rigid segments connected by dynamic β-1,3-glucans [7]. Other polysaccharides (galactomannan, galactosaminogalactan, and β-1,3-1,4-glucan) are covalently bound to the chitin–glucan complex by transglycosidases [8].

In the fungal life cycle, cell wall stress (CWS) occurs in vivo in response to sex pheromones, which induce rearrangements in the CW composition. CWS also arises under various stressors, such as oxidative stress, extreme pH values, DNA-damaging agents, heat shock, and hypo-osmotic shock [3]. Additionally, CWS can be triggered by chemical compounds that disrupt the synthesis or assembly of the primary CW polysaccharides: glucans and chitin. To induce CWS in vitro, azo dyes such as Congo red (CR) and calcofluor white (CFW), lytic enzymes (zymolyase), fungicidal 1,3-β-D-glucan synthase inhibitors (cyclic lipopeptides echinocandins, acidic terpenoids enfumafungins, glycolipids papulacandins), or chitin synthase inhibitors (nikkomycins, polyoxins) are employed [9,10]. These agents weaken the CW and cause fungal cell lysis.

CFW is believed to bind more specifically to chitin, whereas CR predominantly binds to α-1,3- and β-1,3-glucans [11,12]. The addition of CR or CFW to actively growing cells leads to morphological changes, including incomplete separation of maternal and daughter cells in yeast and swelling and lysis of hyphal tips in mycelial fungi. These azo dyes are thought to inhibit the formation of bonds between the newly synthesized chains of chitin, β-1,3-, and β-1,6-glucans, thereby disrupting the secondary structure of the CW, which leads to its weakening and eventual lysis. However, the precise mechanism of action of these azo dyes remains poorly understood. Notably, the effects of CR extend beyond the CW, influencing both the primary and secondary metabolism of *A. fumigatus*, indicating a pleiotropic effect [12].

The cell wall integrity (CWI) pathway is activated in response to CWS, leading to the enhanced synthesis of the main CW polysaccharides—chitin and α-glucan [13]. These findings align with the observed upregulation of gfaA transcription, which encodes glutamine:fructose-6-phosphate amidotransferase, the enzyme catalyzing the first and rate-limiting step in chitin synthesis following CFW-induced CWS in *A. niger* [9]. The Mid-type CWI sensor protein, MtlA, in *A. nidulans* is highly O-glycosylated and localized to the cell surface [14]. Loss of MtlA increases sensitivity to CW inhibitors and reduces CW α-glucan and chitin content. The CWI pathway is a multi-step phosphorylation cascade involving the induction of the GTPases RhoB and RhoD via mitogen-activated protein kinase kinase (MAP2K) and the transcription factor RlmA. However, the sophisticated defense system of *A. niger* employs at least three transcription factors—RlmA, MsnA, and CrzA—under CWS [15]. The CWI pathway plays a crucial role in morphogenesis, virulence, and antifungal susceptibility [16]. However, as observed in yeast, two additional pathways of the general stress response contribute to the response to CWS in aspergilli. These include a transcription factor-mediated pathway analogous to the Msn2p/Msn4p system in yeast, mediated by the MsnA ortholog in aspergilli, and the calcium/calcineurin pathway mediated by Crz1p and CrzA, respectively [17]. These pathways facilitate adaptation to a number of abiotic stressors, such as heat, cold, and osmotic shocks, which involve alterations in the cytosolic osmolyte composition and membrane lipid profiles. Notably, the transcription factor RlmA, which activates the CWI signaling pathway, also affects lipid metabolism, and certain MAP kinases contribute to adaptation not only to CWS, but also to heat and osmotic shocks [17]. In addition, membrane-destabilizing compounds, such as sodium dodecyl sulfate, have been shown to induce CWS. Studies have also revealed interactions among signaling pathways in response to CWS, osmotic, oxidative, and heat shocks [18,19]. These findings raise important questions regarding the roles of osmolytes and membrane lipids in the response to CWS.

Fungi are widely used in biotechnology to produce enzymes, organic acids, and biologically active substances [20]. Most of these products are secreted through the CW. Consequently, mutations in the genes responsible for synthesizing certain CW polysaccharides have been shown to increase the yield of organic acids and enzymes in producer fungi. Additionally, α-1,3-glucan in *Aspergillus* species functions as an adhesive factor involved in pellet formation [21].

Regulation of polysaccharide display on the cell surface allows filamentous fungi to control their macromorphology, such as pellets and loose colonies (floccules), which in turn affects productivity. Additionally, inhibiting CWI signaling is an effective strategy for managing plant and animal pathogens. Fungal CW is a preferred target for developing new antimycotics because its composition differs fundamentally from that of bacterial or plant CW and animal cell membranes [22]. Moreover, differences in the composition and structure between the yeast and filamentous fungi CW may be potentially beneficial. Fungal tolerance to CR has been used as a promising indicator of different ecological niches, such as insect pathogens, plant pathogens, saprotrophs, and mycoparasites [23]. Therefore, studying the response to CWS is of both fundamental and practical importance.

The aim of this study was to investigate the composition of the membrane lipids, osmolytes, and basic polysaccharides of the *A. niger* CW under CWS conditions induced by CR and CFW.

## 2. Results

### 2.1. The Influence of Azo Dyes on A. niger Mycelium Morphology

**Figure 1:**
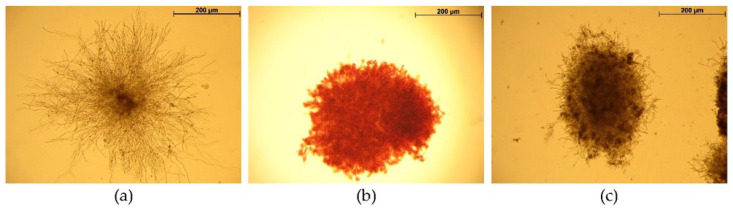
*A. niger* colonies in submerged culture under optimal conditions (**a**) and 4 h after adding Congo red (CR) at a concentration of 50 μg/mL (**b**) or calcofluor white (CFW) at a concentration of 75 μg/mL (**c**). Scale bar is 200 μm.

To develop the experimental protocol, the concentrations of azo dyes were selected (Figure 1). In the control group, after 12 h of cultivation, all spores had germinated and long, unbranched germ tubes had formed. After 16 h, loose colonies (floccules) composed of actively branching germ tubes were observed. Four hours after the application of azo dyes, significant inhibition of apical growth and the formation of swellings in the mycelia were noted compared with the control. Consequently, the following azo dye concentrations were selected: 50 μg/mL for CR and 75 μg/mL for CFW. It is important to note that CR stained the mycelium red; compared with CFW-treated mycelium, numerous bulbous hyphal tips were present, and no apical hyphal growth was observed at the colony edges. In contrast, CFW treatment resulted in significantly fewer hyphal swellings and only weak apical growth at the colony margins. At the same time, no lysis of the apical hyphal tips was observed.

### 2.2. The Influence of Azo Dyes on the Composition of the Main Polysaccharides in the A. niger Cell Wall

**Figure 2:**
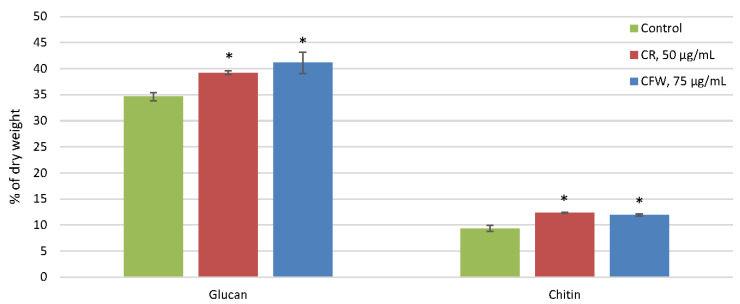
Glucan and chitin content of the cell wall of *A. niger* under CWS, % of dry weight. Means ± SEM are displayed, *n* = 3, SEM—standard error of the mean. Statistically significant difference (*p* ≤ 0.05) is indicated by (*).

In the control, the amount of chitin was significantly lower (9% by weight of CW) than glucan (35%) (Figure 2). The glucan-to-chitin ratio was 3.9. Under the influence of both azo dyes, chitin levels increased by 33%, while glucan levels increased by 11% with CR and by 17% with CFW. Consequently, the glucan-to-chitin ratio decreased to 3.2 for CR and 3.4 for CFW.

### 2.3. The Effect of Azo Dyes on the Composition of A. niger Osmolytes

**Figure 3:**
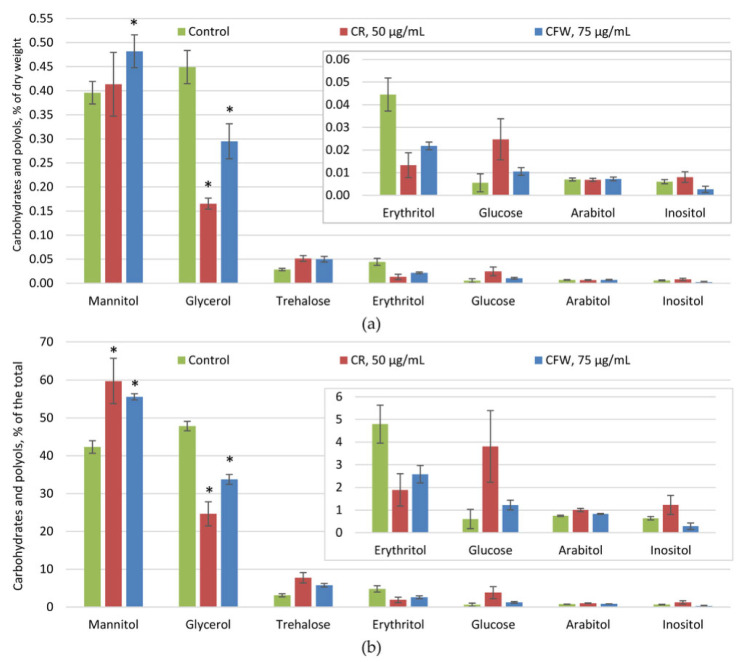
Carbohydrates and polyols of *A. niger* under CWS: (**a**)—% of dry weight; (**b**)—% of the total. Means ± SEM are displayed, *n* = 3, SEM—standard error of the mean. Statistically significant difference (*p* ≤ 0.05) is indicated by (*).

Intracellular soluble sugars (osmolytes) are represented by two dominant polyols—glycerol and mannitol—along with smaller amounts of erythritol and trehalose. Glucose, arabitol, and inositol were present in trace quantities (<0.01% of the dry weight and <3.5% of the total) and did not change significantly under CWS (Figure 3). The amount of osmolytes in the control was approximately 1% of the dry weight, which decreased by half under the influence of CR and by one and a half times in the presence of CFW (Figure 3a). At the same time, the ratios of the main osmolytes changed significantly. In the control, glycerol accounted for about 49% and mannitol for 42% of the total osmolytes (Figure 3b). These proportions shifted markedly under the influence of both azo dyes. Exposure to CR caused a twofold decrease in the glycerol proportion, resulting in a one and a half-fold increase in the mannitol proportion. Under the CFW treatment, the glycerol proportion decreased by one-and-a-half times, while the mannitol proportion increased by 35%. Additionally, the proportion of the minor osmolyte trehalose increased from 3% to 6%.

### 2.4. The Effect of Azo Dyes on the Composition of A. niger Storage and Membrane Lipids and Their Fatty Acids

**Figure 4:**
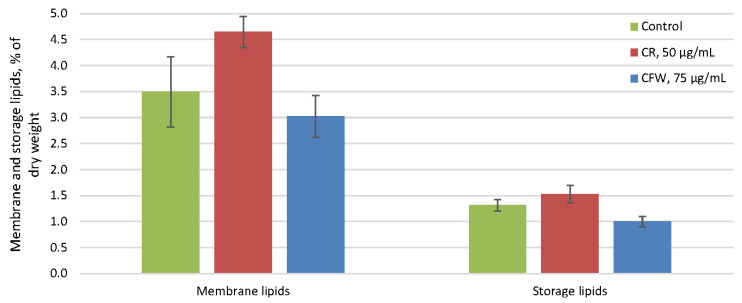
Amounts of membrane and storage lipids (% of dry weight) of *A. niger* under CWS. Means ± SEM are displayed, *n* = 3, SEM—standard error of the mean.

**Figure 5:**
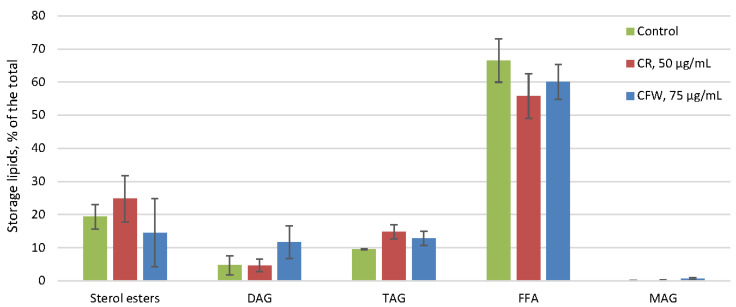
The profile of *A. niger* storage lipids under CWS. Means ± SEM are displayed, *n* = 3, SEM—standard error of the mean. DAG—diacylglycerols, TAG—triacylglycerols, FFA—free fatty acids, MAG—monoacylglycerols.

The amount of the storage lipids in the control did not exceed 1.2% of the dry weight and remained unchanged under the influence of both azo dyes (Figure 4). In the control, storage lipids were predominantly free fatty acids (FFA), comprising 60–65% of the total, with smaller amounts of sterol esters and di- and triacylglycerols (Figure 5). No changes in the composition of the storage lipids were observed in the presence of azo dyes.

**Figure 6:**
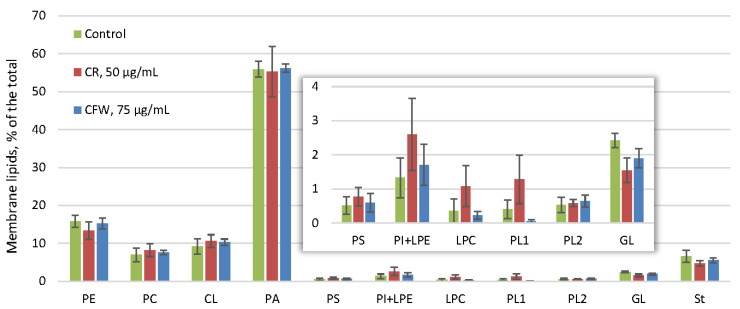
The profile of *A. niger* membrane lipids under CWS. Means ± SEM are displayed, *n* = 3, SEM—standard error of the mean. PE—phosphatidylethanolamines, PC—phosphatidylcholines, CL—cardiolipins, PA—phosphatidic acids, PS—phosphatidylserines, PI—phosphatidylinositols, LPE—lysophosphatidylethanolamines, LPC—lysophosphatidylcholines, PL—unidentified phospholipids, GL—glycolipids, St—sterols.

The amount of membrane lipids in the control reached 3.5% of the dry weight and did not change under the influence of either azo dye (Figure 4). Membrane lipids in the control consisted mainly of phosphatidic acids (PA) (55% of the total), followed by phosphatidylethanolamines (15%), phosphatidylcholines (8%), cardiolipins (10%), and sterols (5%) (Figure 6). Other phospholipids, such as phosphatidylserines, phosphatidylinositols, lysophosphatidylethanolamines, and lysophosphatidylcholines, as well as glycolipids and two unidentified phospholipids, were present in trace amounts. No significant changes in the membrane lipid composition were detected after exposure to either azo dye.

**Figure 7:**
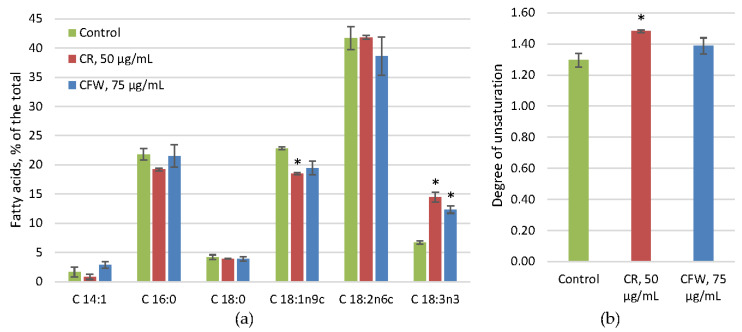
Main fatty acids (**a**) and degree of unsaturation (**b**) of *A. niger* membrane lipids under CWS. Means ± SEM are displayed, *n* = 3, SEM—standard error of the mean. Statistically significant difference (*p* ≤ 0.05) is indicated by (*).

The fatty acids of the phospholipid fraction in the control were predominantly linoleic acid (C18:2), which accounted for 40% of the total. Palmitic (C16:0) and oleic (C18:1) acids each accounted for 20%, with smaller amounts of linolenic (C18:3) and stearic acids (C18:0), and trace amounts of myristoleic (C14:1) acid (Figure 7). Analysis of the fatty acid composition under the influence of azo dyes revealed a general trend of increased linolenic acid accompanied by a decrease in the proportion of oleic acid (Figure 7a). This resulted in an increase in the degree of unsaturation (DU) (Figure 7b). This effect was more pronounced with CR.

## 3. Discussion

The CR and CFW azo dyes used to induce CWS in vitro each contain two sulfonic groups, which are soluble in slightly acidic (pH above 5.6), neutral, and alkaline environments due to the negative charge of the sulfonic groups [11]. To investigate the effects of these dyes, we used Blumenthal–Roseman medium with citrate-phosphate buffer at pH 6.0 to prevent crystallization of CR and CFW, which occurs below pH 5.6 [24]. We preliminarily selected the concentrations of azo dyes using a range of 25, 50, 75, and 100 μg/mL for CR and 30, 50, 75, and 100 μg/mL for CFW. The concentrations of 50 μg/mL for CR and 75 μg/mL for CFW were chosen based on the observed morphological changes: significant growth inhibition and the appearance of hyphal swellings, without lysis of the apical hyphal tips. In the experiment, CR and CFW were added to a 12 h submerged culture after all spores had germinated and formed long, unbranched germ tubes. After 4 h, loose colonies composed of actively branching and growing hyphae were observed in the control. Under the influence of the azo dyes, apical growth was inhibited, and numerous hyphal swellings formed. In the CR-treated variant, there were more bulbous swellings and near-complete arrest of apical growth, whereas in the CFW-treated mycelia, fewer swellings were observed and growth was slowed down (Figure 1). The presence of hyphal swellings aligns with the data indicating that CR and CFW disrupt microfibril formation, thereby compromising CW integrity. This disruption stimulates chitin synthesis, leading to its localization not only in the inner layer but throughout the entire width of the CW. Due to impaired microfibril formation, chitin no longer provides rigidity to the CW [5]. Analysis of CW polysaccharides in *A. niger* revealed that these morphological changes induced by azo dyes were accompanied by increases in both the chitin and glucan levels (Figure 2). An increase in chitin content is a well-established marker of CWS [9], while an increase in glucan has only been reported in a few studies so far [25]. Interestingly, CR stress causes only minor changes in the CW composition of *A. nidulans* [26]. It is known that chitin constitutes no more than 30% of the CW in aspergilli, whereas the glucan content ranges from 30% to 80% [5,6]. During the ontogenesis of *A. niger*, chitin is more prevalent in the chitin–glucan complex of mature spores and conidiophores, while glucan predominates within idiophase mycelia [27]. The major components of the *A. fumigatus* CW include β-1,3-glucans, α-1,3-glucan, chitin, and a mixed unique β-1,3-/β-1,4-glucan [4]. In the CW of the studied *A. niger* strain, the glucan content was three times higher than that of chitin (Figure 2). However, under the azo dye treatment, chitin content in the CW of fungal germlings increased more significantly (by 33%) compared to glucan (which increased by 11–17%). Both azo dyes exert similar effects. The mechanism underlying growth arrest remains unclear; however, evidence indicates that CR, which induces more pronounced morphological changes, exerts a pleiotropic effect [12]. Unlike CFW, CR completely inhibits polarized growth and leads to the formation of large Quasimodo cells when added during spore inoculation. Although comparable data characterizing the effects of CFW are not yet available, it is evident that their mechanisms of action differ, consistent with our findings—particularly regarding the numerous hyphal swellings observed.

Osmolytes, primarily including trehalose and polyols in fungi [28,29,30], are known to play significant roles in adaptation to various abiotic stressors and contribute substantially to fungal thermophilia [31], alkalophilia [32], acidophilia [33,34], xerophilia [35], and psychrophilia [36]. However, analysis of the osmolyte composition in the studied fungus revealed that osmolytes were not involved in the adaptation to CWS induced by CR and CFW, as their levels did not increase. Notably, in the control, the osmolyte content in the germlings was low (below 1% of dry weight), with osmolytes comprising comparable proportions of glycerol and mannitol (Figure 3). Exposure to CR and CFW reduced the total osmolyte levels, mainly due to a decrease in glycerol concentration, which lowered its proportion from 49% to 25–35%, while the proportions of mannitol and trehalose increased from 42% to 55–60% and from 3% to 6–8%, respectively (Figure 3b). Previous studies have shown that carbohydrates and polyols (CaP) can reach 8–9% of dry weight in a 24 h submerged culture, with mannitol amounting to 70% of the total [37]. Glycerol is known to play a crucial role in maintaining turgor pressure during the early stages of fungal spore germination, when the CW is newly formed and much thinner than in mature cells [5]. This is exemplified by the germination of spores from the citric acid producer *A. niger* P-36-09-00 [38]. Resting spores contained about 13% CaP by dry weight, predominantly mannitol (41%) and trehalose (31%), with glycerol comprising no more than 6%. At the initial germination stage (4 h, swelling), CaP content decreased threefold, accompanied by a dramatic shift in composition: glycerol proportion increased to 45%, while mannitol and especially the trehalose proportions decreased. After 12 h of germination (onset of sprout tube branching), mannitol (48%) and glycerol (33%) were the main CaP, with the trehalose level remaining below 10%. During growth, the CWI pathway has been implicated in monitoring the thickness and elastic properties of the CW [5]. It has been shown that in *Schizosaccharomyces pombe*, polarized growth domains of the CW are thinner and softer, and that increasing the wall thickness negatively influences growth [39]. In this context, turgor pressure—generated by the osmotic expansion of the cell membrane pushing against the CW—may be weakened by the accumulation of osmolytes in the cytoplasm [29,38]. Based on these data, it can be suggested that in the studied *A. niger* strain, the decrease in glycerol levels correlates with increased CW strength, likely due to elevated chitin content, which may also help maintain turgor pressure. Interestingly, exposure to azo dyes led to increases in both the amount and proportion of trehalose in the fungus; however, its overall content remained very low (below 0.05% of dry weight) (Figure 3a). This low concentration suggests that trehalose does not provide a significant protective effect, despite being a multifunctional compound with protective, antioxidant, chaperone, transport, and storage functions [40,41,42,43,44,45].

The cytoplasmic membrane and the CW together constitute the cell envelope [1]. Many processes involved in the synthesis and assembly of CW components occur within membranes. The composition of the membrane lipids of the *A. niger* mycelium in the control was unusual: the predominant phospholipid were the non-bilayer PA, comprising about 55% of the total lipids. In contrast, the bilayer PC and non-bilayer PE, which are typically characteristic of fungal membrane lipids, accounted for only about 8% and 15%, respectively. The proportion of sterols did not exceed 5% (Figure 6). Previous studies showed that in a 24 h submerged culture, the proportion of PA was only 15%, while the proportions of PC and PE increased to 20% each [37]. Exposure to both azo dyes did not alter the membrane lipid composition (Figure 6).

The predominance of PA in the composition of membrane lipids during the early stage of *A. niger* growth was observed for the first time. PA are multifunctional compounds [46] and serve as key intermediates in the synthesis and exchange of phospholipids and storage lipids. Due to the conical shape of the PA molecule and its tendency to aggregate, PA microdomains can induce membrane curvature, which determines their roles not only in membrane structure, fusion, and division but also in membrane trafficking, endocytosis, and exocytosis [47,48,49]. Another important function of PA is signaling, which involves specific binding to proteins. The signaling function of PA is closely related to its unique structure and physicochemical properties. The anionic phosphate head group of PA carries two negative charges, which enables it to interact with proteins or form hydrogen bonds more readily than other anionic phospholipids [50]. PA has emerged as a novel class of lipid mediators that influence membrane structure, dynamics, and protein interactions. For example, PA has been reported to regulate the transcriptional repressor Opi1p, which is involved in phospholipid metabolism [51]. As a secondary messenger, PA regulates processes in eukaryotic cells such as endocytosis, exocytosis, membrane trafficking, cytoskeleton formation, organelle contact and dynamics, growth and metabolism, and programmed cell death [50]. A high relative content of PA in membrane lipids has been reported under abiotic stress and in extremophiles [31,32,33,35,36,52]. Given the multifunctionality of PA, it is challenging to pinpoint its specific role during the active growth phase of *A. niger*. However, it is clear that the elevated PA content in the membrane lipids is not associated with CWS, as the amount and proportion of PA do not change under exposure to azo dyes. Apical growth in fungi requires vesicle-mediated transport of molecules essential for CW biosynthesis and plasma membrane expansion [53]. It can be assumed that, besides its signaling function, the high PA proportion in membrane lipids of *A niger* likely reflects the active synthesis of phospholipids and membrane vesicle trafficking toward the hyphal tip.

Despite the constant overall composition of membrane lipids, analysis of the fatty acid composition of phospholipids revealed an increase in the proportion of linolenic acid (C18:3) and a decrease in oleic acid (C18:1) under the influence of CR and CFW (Figure 7a), resulting in an increased DU of phospholipids (Figure 7b). Meanwhile, the proportion of linoleic acid (C18:2) (approximately 40%) remained unchanged. It is well-established that membrane fluidity is influenced by the DU of phospholipid fatty acids [54], the sterol-to-phospholipid ratio [55], and the presence of small heat shock proteins [56]. In the studied fungus, the overall membrane lipid composition did not change; consequently, the sterol-to-phospholipid ratio remained constant in the presence of azo dyes. However, the increased proportion of polyunsaturated linolenic acid and the resulting rise in DU may contribute to the enhanced membrane fluidity. The effects of fatty acid composition on membrane lipid properties and growth have yet to be fully explored [57]. For example, in *Aspergillus nidulans*, simultaneous deletion of sdeA and sdeB, which encode Δ9-fatty acid desaturases, completely abolishes growth [58]. Deletion of odeA, encoding a Δ12-fatty acid desaturase, results in a slight decrease in colony size and a significant reduction in conidia production [59], whereas deletion of odeB, encoding a Δ15-fatty acid desaturase, has only a minor impact on growth [57]. In the studied fungus, during active growth, approximately 70% of the fatty acids in phospholipids were unsaturated (Figure 7). Under the influence of CWS, the proportion of the highly unsaturated linolenic acid increases, suggesting its involvement in the adaptation process; however, further research is needed to clarify its specific function.

The mechanism of action of azo dyes on fungal cells remains poorly understood. However, early studies indicate that azo dyes do not affect a single gene associated with the synthesis of a specific polysaccharide but instead exert a pleiotropic effect. For example, in *A. fumigatus*, CR stimulated the expression of 432 genes and suppressed the expression of 271 genes, even at a low concentration of 50 μg/mL [12]. Moreover, CR did not affect the genes responsible for synthesizing the main CW polysaccharides—chitin and β-1,3-glucan—but stimulated the synthesis of N-acetyl-D-glucosamine and N-acetyl-D-galactosamine. Increased expression of genes involved in the reorganization of β-1,3-glucan and α-1,3-glucan was also observed. In addition to its effects on the CW, azo dyes increased the expression of genes related to primary metabolism, with the strongest impact on the synthesis of secondary metabolites. Our studies have shown that protective mechanisms such as osmolyte and membrane systems are not involved in the adaptation to CWS caused by azo dyes, despite previous assumptions based on studies of CWS response regulation pathways [17,18,19]. It was revealed that CWS occurs under the influence of various stressors—including azo dyes, fungicides, inhibitors of polysaccharide synthesis, zymolyase, etc.—that induce changes in CW composition, and the mechanisms underlying their effects may differ. Transcriptomic data indicate that, in addition to the transcription factor RlmA, MsnA and CrzA are also important for *A. niger* to withstand CWS [15]. Thus, the CWI pathway in *A. niger* is not the sole compensatory mechanism involved in repairing the compromised CW. It has been shown that RlmA is the primary transcription factor required for protection against CFW, but it cooperates with MsnA and CrzA to ensure the survival of *A. niger* when challenged with caspofungin and aureobasidin A. Furthermore, RlmA stimulates the expression of genes involved not only in chitin and glucan synthesis but also in lipid metabolism, suggesting a link between changes in the CW and membrane lipids. This relationship between lipid metabolism and the CWI pathway has been confirmed, as treatment with drugs that disrupt CWI results in the altered expression of lipid biosynthesis enzymes in wild-type strains or mutants of the CWI or high-osmolarity glycerol pathways [53]. However, our data show that in the case of CWS caused by azo dyes, the composition of membrane lipids did not change. This may be because azo dyes do not inhibit polysaccharide synthesis but rather stimulate it. It is possible that in the case of inhibitors of chitin and glucan synthesis, changes in membrane lipids would also be observed.

The increase in chitin synthesis is a central response to CWS. We have demonstrated that this response reliably protects the cell from stress without causing changes to the membranes or the osmolyte system. This finding underscores the need to develop new inhibitors targeting chitin and glucan synthesis—the primary components of the fungal CW—to combat pathogenic fungi. Another promising approach is to identify inhibitors that prevent cells from activating their CWI defense system, thereby leading to cell death. Additionally, this discovery can be leveraged to accumulate chitin in fungal biomass, which may have biotechnological applications. For example, a mutation in the gene encoding the negative transcription elongation regulator cwcA increased the chitin content in the CW of *A. niger* by 40%, enabling the utilization of waste from enzyme and organic acid production based on this fungus to develop biotechnological processes for producing fungal chitin [25].

Thus, the action of CR and CFW leads to significant changes in the morphology of *A. niger*, including the inhibition of apical growth and the formation of hyphal swellings, an increase in the amount of chitin and glucan in the CW, a decrease in cytosolic glycerol levels, and an increase in the DU of phospholipid fatty acids. Meanwhile, the amount and composition of the membrane and storage lipids remained unchanged. Taken together, these results indicate that the cell’s osmolyte and membrane systems are largely uninvolved in the response to CWS induced by CR and CFW. The minor changes observed in osmolyte and membrane composition may be associated with the strengthening of the CW due to an increased polysaccharide content.

The results obtained in this study do not exclude the possibility that other stressors causing CWS may affect the cell membrane and osmolyte systems. We plan to further investigate this issue in two ways: by using inhibitors of chitin and glucan synthesis to induce CWS and by determining whether abiotic stressors, such as heat and osmotic shock, also trigger CWS.

## 4. Materials and Methods

### 4.1. Objects of Study and Cultivation Protocol

Object of the study was ascomycete filamentous fungus *Aspergillus niger* van Tieghem 1867 VKM F-34, (*Aspergillaceae*, *Eurotiales*, *Eurotiomycetidae*, *Eurotiomycetes*, *Pezizomycotina*, *Ascomycota*, *Fungi*).

The fungus was cultivated at the optimal temperature of 29–30 °C for 5–6 days on slanted wort agar (3.5° Balling). For submerged cultivation, a spore suspension was added to achieve a concentration of 5 × 10^5^–10^6^ spores/mL in the buffered Blumenthal–Roseman medium [60]. The liquid medium consisted of two components: (1) a citrate-phosphate buffer, prepared with 0.1 M citric acid and 0.2 M Na_2_HPO_4_ adjusted to a final pH value of 6.0, and (2) the nutrient component (described below), adjusted to pH 6.0 using a 33% NaOH solution. The components were prepared separately and mixed in 1:1 ratio, resulting in a final pH value of 6.0, and the following nutrient concentrations in the medium (g/L): sucrose—30, KH_2_PO_4_—10, (NH_4_)_2_SO_4_—10, MgSO_4_ × 7H_2_O—1, KCl—0.5, yeast extract—0.3, FeSO_4_ × 7H_2_O—0.01, ZnSO_4_ × 7H_2_O—0.05.

Submerged cultivation of the fungus was carried out in 250 mL flasks containing 50 mL of the medium on KЭ-12-250T shakers (Moscow, Russia) at 150 rpm. Cultivation was performed at the optimal temperature of 29–30 °C for 12 h. Azo dyes were then added to the flasks at final concentrations of 50 μg/mL for CR (Aldosa, Moscow, Russia) and 75 μg/mL for CFW (Himedia, MH, India), and cultivation continued for an additional 4 h. Control was maintained under the same conditions without the addition of azo dyes.

For biochemical analyses, the fungal mycelium was separated from the culture fluid using a nylon filter, then washed with distilled water. Excess moisture was removed, and the biomass was weighed and stored at −21 °C. Dry biomass was determined gravimetrically.

### 4.2. Lipids, Carbohydrates, and Polyols Analysis

For the analysis of lipid composition, a weighed sample of wet biomass was homogenized in isopropanol and extracted for 30 min at 70 °C, and the supernatant was decanted. The precipitate was then extracted twice with a 1:1 mixture of isopropanol and chloroform, followed by one extraction with a 1:2 mixture of the same solvents under the same conditions (modified Nichols method) [61,62]. The combined extracts were dried using a rotary evaporator, and the residue was dissolved in 9 mL of a 1:1 chloroform–methanol mixture. To this, 12 mL of 2.5% NaCl solution was added to remove water-soluble compounds. After phase separation, the chloroform layer was collected, dried over anhydrous Na_2_SO_4_, and concentrated using a vacuum rotary evaporator. The residue was then dried to a constant mass under vacuum. Finally, the residue was dissolved in a 2:1 chloroform–methanol mixture and stored at −21 °C. Lipids were separated using two-dimensional (polar lipids) or one-dimensional (neutral lipids) thin-layer chromatography (TLC) [63,64] and quantified using standard compounds by the densitometry method (DENS software, version 5.1.0.2). To study the composition of fatty acids, the polar lipid fraction was isolated using one-dimensional TLC. The polar lipid spots at the start were scraped out and eluted with a mixture of chloroform:methanol (1:1), then the extract was evaporated and methanolysis was carried out using 2.5% H_2_SO_4_ in methanol for 2 h at 70 °C. The obtained methyl esters were analyzed by gas–liquid chromatography (GLC) on a Kristall 5000.1 gas–liquid chromatograph (Chromatec, Yoshkar-Ola, Russia) with an Optima-240, 60 m × 0.25 µm × 0.25 mm capillary column (Macherey-Nagel GmbH&Co, Düren, Germany). The temperature program used was from 130 °C to 240 °C at a rate of 5–6 °C/min. Identification was carried out using the Supelco 37 Component FAME Mix mixture of individual fatty acid methyl esters (Supelco, Bellefonte, PA, USA). The DU of the phospholipids was calculated according to the following equation [65]:

$$DU=\frac{1.0\times \left(\%\;monoene\;FA\right)}{100}+\frac{2.0\times \left(\%\;diene\;FA\right)}{100}+\frac{3.0\times \left(\%\;triene\;FA\right)}{100}+\frac{4.0\times \left(\%\;tetraene\;FA\right)}{100}$$

To determine the soluble carbohydrate composition of the mycelium, sugars were extracted with boiling water for 20 min four times. Proteins were removed from the resulting total extract [66]. The carbohydrate extract was further purified from charged compounds using a combined column with the Dowex-1 (acetate form) (Merck, Darmstadt, Germany) and Dowex 50 W (H^+^) (Merck, Darmstadt, Germany) ion exchange resins. Carbohydrate composition was determined by GLC using trimethylsilyl sugar derivatives obtained from the lyophilized extract [67]. The internal standard was α-methyl-D-mannoside (Merck, Darmstadt, Germany). Chromatography was carried out on a Kristall 5000.1 gas chromatograph (Chromatec, Yoshkar-Ola, Russia) with a ZB-5 30 m, 0.32 mm, 0.25 μm capillary column (Phenomenex, Torrance, CA, USA). The temperature was increased from 130 °C to 270 °C at a rate of 5–6 °C/min. Glucose, mannitol, arabitol, inositol, glycerol, erythritol, and trehalose (Sigma, St. Louis, MO, USA) were used as standards.

### 4.3. Cell Wall Isolation

Fungal CW was isolated following the modified Ram et al. method [9]. Approximately 1.5 g of crude mycelium was ground using a mortar and pestle, with the degree of disruption monitored under a Jenaval microscope (Carl Zeiss, Jena, Germany). Then, 20 mL of 1 M NaCl was gradually added, and the mixture was heated at 100 °C for 5 min to inactivate enzymes. The mixture was centrifuged at 5000 rpm, and the residue was extracted three times with 1 M NaCl, followed by three extractions with 20 mL of distilled water to remove intracellular contents. The remaining residue was treated with ethanol, centrifuged at 5000 rpm, and subsequently freeze-dried. The purification of the CW was monitored under a microscope with a magnification of 250× and 400×.

### 4.4. Analysis of Chitin and Glucan

The amount of chitin in the fungal CW was determined by measuring glucosamine using the Boas method with para-dimethylaminobenzaldehyde (CDH, New Delhi, India) [68], following the hydrolysis of 10 mg of CW with 10 mL of 6 N HCl for 6 h at 100 °C.

The amount of glucan in the CW was determined by analyzing the sugar content using the phenol-sulfuric acid method [69]. A hydrolysate was prepared by treating 10 mg of CW with 10 mL of 3 N HCl for 3 h at 100 °C.

### 4.5. Statistical Analysis

The experiments were carried out in triplicate, *n* = 3. The post hoc Dunnett test was used for pairwise comparison between Control and CR or CFW. On all graphs, mean values ± SEM (standard error of the mean) are plotted. Statistically significant difference (*p* ≤ 0.05) is indicated by (*).

## Data Availability

The original contributions presented in this study are included in the article. Further inquiries can be directed to the corresponding author.
